# Preventing the rejection of skin allografts by immunomodulatory and regenerative effects of exosomes derived from bone marrow mesenchymal stem cells in mice

**DOI:** 10.22038/ijbms.2025.82564.17841

**Published:** 2025

**Authors:** Parham Soufizadeh, Gholamreza Nikbakht Brujeni, Mohammad Mehdi Dehghan, Massoumeh Jabbari Fakhr, Pouya Houshmand, Mahyar Mohebbi, Hossein Aminianfar, Sirous Sadeghian Chaleshtori

**Affiliations:** 1 Biomedical Research Institute, University of Tehran, Tehran, Iran; 2 Gene Therapy Research Center, Digestive Diseases Research Institute, Shariati Hospital, Tehran University of Medical Sciences, Tehran, Iran; 3 Department of Microbiology and Immunology, Faculty of Veterinary Medicine, University of Tehran, Tehran, Iran; 4 Department of Surgery and Radiology, Faculty of Veterinary Medicine, University of Tehran, Tehran, Iran; 5 Department of Tissue Engineering and Applied Cell Sciences, School of Medicine, Qom University of Medical Sciences, Qom, Iran; 6 Department of Pathology, Faculty of Veterinary Medicine, University of Tehran, Tehran, Iran; 7 Department of Internal Medicine, Faculty of Veterinary Medicine, University of Tehran, Tehran, Iran

**Keywords:** Exosome, Immunomodulation, Mice, Mesenchymal stem cells, Regeneration, Transplantation

## Abstract

**Objective(s):**

Reducing the immune response to inflammation is vital for successful transplantation, yet chronic graft rejection remains a major issue despite immunosuppressive drugs. This study explored the effect of bone marrow mesenchymal stem cell-derived exosomes on the survival of skin allografts in mice.

**Materials and Methods:**

C57BL/6 and BALB/c mice underwent skin allograft surgery, followed by intraperitoneal injection of exosomes, which were compared with groups receiving dexamethasone and no treatment group.

**Results:**

On day 3, mild signs of graft rejection appeared in both control groups, while none were seen in the exosome-treated group. By day 14, the grafts were completely rejected in the control groups but showed mild rejection in the treatment group. Histopathology revealed severe rejection signs in the control groups, including epithelial necrosis and inflammation, while the treatment group showed signs of angiogenesis and graft acceptance. Additionally, inflammatory cytokine levels (TNF-α, IL-1β, and IL-6) were lower in the treatment group than in the positive control group, particularly on days 3 and 14.

**Conclusion:**

The findings suggest that exosomes can prevent graft rejection and may offer a promising therapeutic approach for solid organ transplantation, though further research is needed to standardize exosome methods and evaluate cost-effectiveness.

## Introduction

Minimizing the immune response stands as a paramount and indispensable objective for optimizing post-transplantation outcomes. The ideal form of immunosuppression is imperative for ensuring the enduring viability of the graft over an extended period. Over the past decades, diverse chemicals and pharmaceutical agents have been formulated to attenuate acute and chronic graft rejection, with their mechanisms of action exhibiting substantial similarity (1, 2). Nevertheless, despite deploying these pharmacological interventions, the incidence of chronic graft rejection persists at elevated levels (3–5). Moreover, prolonged usage of these agents has been associated with adverse effects such as renal impairment, neuropathy, and heightened susceptibility to adverse events, among others (6, 7). These challenges underscore the pressing necessity to explore superior strategies for immunosuppression and graft preservation (8, 9). Despite the considerable advancements in the realm of pharmaceutical science, there remains a conspicuous absence of suitable drugs dedicated to preventing graft rejection (4). Such an agent should ensure the enduring survival of the graft while exhibiting minimal adverse effects.

Transplantation stands as the preferred treatment for irreversible organ damage (10, 11), yet transplant rejection poses a substantial hurdle in this therapeutic process. Transplant rejection can manifest in subacute, acute, and chronic forms, all characterized by deleterious immune responses necessitating suppression (12). A crucial consideration in the context of solid organ transplant rejection is the ability to anticipate the timing and intensity of the immune response during rejection episodes. It is well-established that controlling an ongoing immune response is considerably more challenging than preventing its initiation (13, 14). Therefore, the current therapeutic approach predominantly focuses on preemptive measures to avert transplant rejection.

Mesenchymal stem cells (MSCs) are increasingly recognized as a valuable asset in the field of regenerative medicine, thanks to their distinct features, notably their capacity to modulate the immune system. MSCs can modulate and suppress the immune response by inhibiting various immune cells through direct cell-cell contact or paracrine secretory factors (15). The secretome of MSCs, including exosomes, has shown immunomodulatory effects on the innate and adaptive immune systems (16, 17). Exosomes are extracellular vesicles (EVs) that are crucial in intercellular communication. They contain various components, such as mRNAs, microRNAs, growth factors, cytokines, and chemokines, which contribute to their therapeutic effects (18, 19). MSC-derived EVs consist of microvesicles (ranging from 0.1 to 1 mm in diameter) and exosomes (measuring between 40 and 100 nm in diameter). These EVs play crucial roles in angiogenesis, tissue regeneration, and immune regulation by ferrying paracrine factors and facilitating micro-communication within the interstitial space. Exosomes possess cell tissue-specific homing abilities, allowing them to convey bioactive molecules to their intended target sites. Reports suggest that the mechanism by which MSCs promote tissue repair is primarily associated with their paracrine functions rather than cellular differentiation. As a result, exosomes have found application in a variety of therapies for tissue damage. (20). Recent studies have investigated the regenerative effects of exosomes derived from MSCs in models of acute kidney injury, liver injury, and myocardial ischemia-reperfusion injury (5, 18) and also skin repair and regeneration (21). 

In this study, we aim to explore the impact of exosomes derived from MSC on the recipient mice’s immune response towards allograft-transplanted tissue. Our investigation will not only delve into the immunomodulatory properties of these exosomes but also assess their regenerative capabilities on the transplanted tissue cells. Amidst the ongoing debate surrounding exosomes, they emerge as a promising candidate for our research objectives. A limited body of research exists investigating the direct influence of exosomes on organ transplantation, with most prior studies primarily focused on inducing tolerance to transplanted tissue. However, our study seeks to probe the direct effects of mesenchymal stem cell exosomes on graft enhancement. The field of tissue regeneration is a dynamic and evolving area of human knowledge, albeit with its persistent challenges. Thus, the utilization of exosomes as a means to achieve these objectives holds promise as a potential solution to address these issues.

## Materials and Methods

### Extraction and characterization of extracellular vesicles (EVs)

*Cell culture and characterization*

MSCs were isolated from the bone marrow of male BalbC mice, aged 6 to 8 weeks, following the methodology outlined in Nadri *et al.* in 2007 (22). The cells were cultured and differentiated into osteogenic and adipogenic lineages according to established protocols. Cell cultures were maintained until the third passage, and the medium from this passage was stored at -80 °C. Both morphological and differentiation assays were employed to characterize the MSCs. Daily observations under an inverted light microscope assessed cell adhesion and morphology. To further verify differentiation, cells underwent staining with Alizarin Red S for osteogenesis and Oil Red O for adipogenesis, in addition to morphological examination. Cells were also harvested and analyzed via flow cytometry to evaluate CD34, CD45, CD73, and CD105 marker expression.

*Exosome extraction and characterization*

Exosomes were isolated using AnaCell’s exosome extraction kit (Anaexo™), following the manufacturer’s protocol, with samples stored at -80 °C. For characterization, samples were examined using a scanning electron microscope. 

### Skin graft surgery and treatment

*Animals*

Female C57BL/6 and BALB/c mice, aged 7–8 weeks and weighing 17–22 grams, were sourced from Razi Vaccine and Serum Research Institute® and housed in the animal facility of the Faculty of Veterinary Medicine, Tehran University. Experimental procedures were approved by the university’s Institutional Laboratory Animal Care and Use Committee, and measures were taken to reduce animal usage. The mice were group-housed, with 3–5 animals per cage, and randomly assigned to three groups: a Negative Control group (undergoing surgery without further treatment to observe natural skin transplantation responses), a Positive Control group (treated with dexamethasone), and a Treatment group (treated with Exosomes). Female mice were selected because female mice generally exhibit lower aggression than males, reducing stress-induced variability in healing studies and supporting more reliable experimental outcomes (23).

*Surgery procedure*

Mice were given a mixture of Ketamine hydrochloride at a dosage of 100 mg/kg and Xylazine at a dosage of 8 mg/kg, which was injected intraperitoneally. The hair in the lumbar area (surgical site) was removed by using clippers and depilatory cream. Then, the area was washed with a sodium chloride solution. A solution of povidone-iodine and alcohol was applied to the area. The donor mice were euthanized, and immediately after euthanasia, the surgical procedure for skin removal was performed within a time frame of approximately 15 to 20 min. The harvested skin was rinsed with normal saline and cut into circular pieces using an 8 mm skin punch. The skin pieces were then placed in a sterile petri dish with moistened sterile gauze with normal saline and stored at 4 °C for a maximum of 60 min (24–26). The recipient mice’s skin defect was created using a 6 mm skin punch and curved scissors, and the panniculus carnosus layer was entirely removed. The graft skin tissue is then placed in the defect site, with the epidermal layer positioned outward. The graft was stabilized by 8 sutures by interrupted stitches using 6–0 Vicryl. It should be noted that different recipient mouse strains were used compared to the donor mouse strains for the grafts. Specifically, when graft skin from the BALB/c strain was obtained, it was transplanted onto C57BL/6 mice, and vice versa. After surgery, mice were placed under observation in a separate space for one hour. Additionally, 0.07 mL Meloxicam (1/10 diluted) was injected subcutaneously for pain relief. For bandaging, sterile Vaseline gauze was prepared according to the size of the wound and placed in the area and was fixed by a layer of transparent adhesive dressing, Tegaderm™, and multiple layers of adhesive bandage tape (24).

*Post-operative care*

The vital status of the mice was assessed daily, and to promote their behavioral health, all mice were encouraged to consume rewarding food items such as fruits, cheese, and sweet cakes. The drug treatment process was carried out in both the positive control group and the treatment group. In the treatment group, exosomes were administered intraperitoneally on days 0 (surgery day) and 2 at a dosage of 100 µg in 0.1 ml. Dexamethasone was administered in the positive control group at a dose of 0.6 mg/kg and intramuscularly on day 1. 

### Graft analysis

*Histopathological and macroscopic analysis of allografts*

Sampling was conducted in two-time intervals. Each group was divided into two subgroups for sampling on days 3 and 14. In each time interval, the graft site was visually examined. Imaging was performed to document the visual characteristics of the site. The graft sites were examined in terms of size (>50% reduction in size), color (dark blue, brown, black), and texture (stiffness) (24). Skin tissue samples were collected from the graft sites, fixed with paraformaldehyde, and stained with H&E. Tissues were analyzed by a pathologist blinded to the study. 

*Real-time PCR*

Spleen tissue was used for immunological tests. To assess the immunomodulatory effects of the intervention on the treatment group and the positive control group, the expression levels of inflammatory cytokines genes such as interleukin-1 beta (IL-1β), interleukin-6 (IL-6), and tumor necrosis factor-alpha (TNF-α) were measured by using the relative quantitative Real-Time PCR technique. The beta-actin gene was used as a housekeeping gene, and the negative control group was used as the calibrator (27). The primer sequences for the mentioned genes were obtained from the information provided on the OriGene^©^ company website (https://www.origene.com) (Table 1). The iNtRON^®^ extraction kit was used for RNA extraction. After RNA extraction, a commercial kit (Takapuzist™ company, Iran) was used for generating cDNA. Finally, the samples were stored and preserved at -80 °C.

The Real-Time PCR technique was performed by an initial denaturation step at 95 °C for 5 min, followed by 35 cycles of 30 sec at 95 °C, 30 sec at 60 °C, and 30 sec at 72 °C. For data normalization and obtaining the final concentration, several standard concentrations of the beta-actin gene were examined, and the resulting graph was plotted.

### Statistical analysis

Data was processed in Excel to calculate ∆CT, ∆∆CT, and Fold Change by using the Livak method (27). The statistical analysis was conducted utilizing GraphPad Prism 9.5.0, with statistical distinctions assessed through either ANOVA and Tukey’s *post hoc* test or the unpaired t-test. The results are presented as mean ± standard deviation (SD), and *P*-values less than 0.05 were deemed statistically significant.

## Results

### Cells and extracellular vesicles

*Mesenchymal stem cells*

The morphology of MSCs is characterized by a spindle shape with a small cell body and long, stretched cell extensions. The cell body contained a large, round, and prominent nucleus, exhibiting a bright appearance. These features were consistently observed in the flask samples. Upon exposure to a bone differentiation medium, MSC transformed bone stem cells, specifically osteoblasts. These differentiated cells exhibited a larger size and a star-shaped appearance. Additionally, the presence of adipose cells was observed in the adipogenesis medium. Flow cytometry analysis revealed that more than 90% of MSC lacked the hematopoietic markers CD34 and CD45, indicating their non-hematopoietic nature. Furthermore, the surface markers CD73 and CD105, specific to MSC, were presented in a major part of the cells.

*Extracellular vesicles*

**Figure 1:**
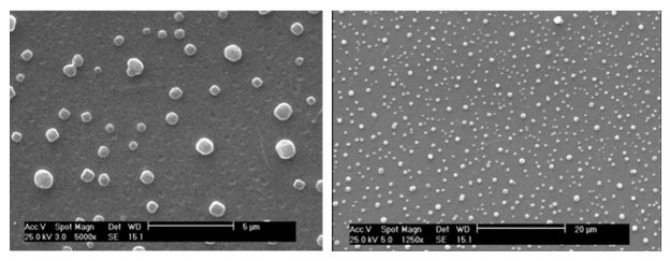
Images prepared by scanning electron microscope with 2 different magnifications

Scanning electron microscopy of the samples showed the presence of numerous spherical bodies with relatively uniform and homogeneous structures. Based on their size and characteristics, these particles were identified as small extracellular vesicles, specifically exosomes (Figure 1). The derived exosomes were administered intraperitoneally at a concentration of 100 µg in a volume of 0.1 ml for each treatment course.

### Efficacy results

*Visual observations*

**Figure 2:**
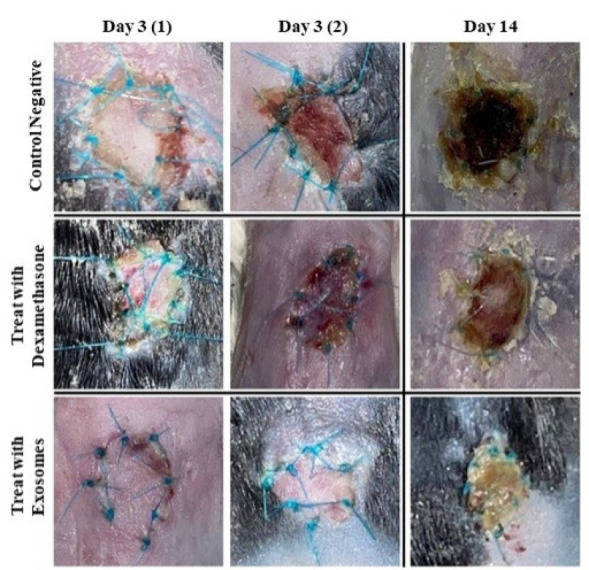
Transplantation outcome in mice at different time points (Days 3 and 14)

Visual observations of the graft tissue in the negative control group on day 3 revealed browning of the tissue along with a completely hard and compact texture. Similar conditions were observed in the positive control group, although the severity of graft rejection symptoms was comparatively less. On day 3, minimal signs of graft rejection were present in the treatment group. By day 14, all groups exhibited signs of graft rejection, with the negative and positive control groups showing complete rejection characterized by black tissue color and a hard, compact texture. In contrast, the treatment group displayed milder symptoms, such as tissue browning and a semi-hard texture (Figure 2). No significant differences were observed in the response to exosome treatment between BALB/c skin grafts transplanted onto C57BL/6 mice and vice versa. Comparable outcomes were yielded in both setups, indicating that the efficacy of exosome therapy was not affected by the donor skin strain.

*Histopathology*

**Figure 3:**
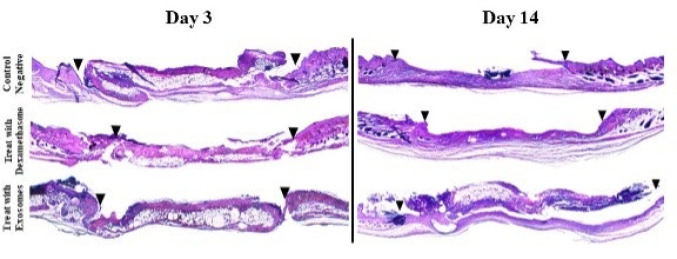
Scanned images of pathology slides from mice on Days 3 and 14, stained with hematoxylin and eosin (H&E)

**Figure 4:**
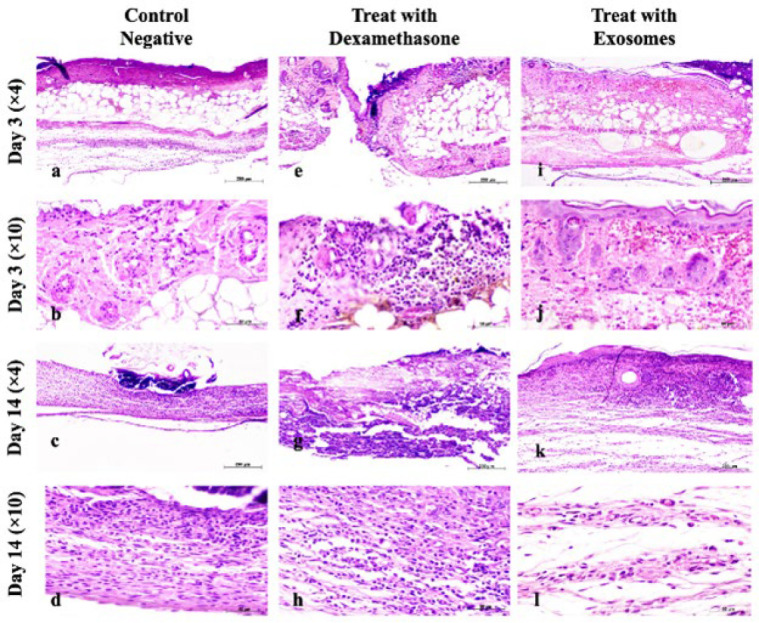
Histopathology of transplanted grafts in mice different time points (Days 3 and 14), stained with hematoxylin and eosin (H&E)

Qualitative examination of pathological sections obtained on days 3 and 14 provided further insights. On day 3, acute graft rejection signs were observed in the negative and positive control groups, including epithelial necrosis, fat tissue presence, muscular layer necrosis, hair follicle necrosis, and infiltration of inflammatory cells (28–32). Bacterial colonies were also detected in the negative control group, while brown regions resulting from the degradation of red blood cells were observed in the positive control group. The treatment group exhibited healthy epithelium, along with minor hemorrhage and infiltration of inflammatory cells, although the severity of these signs was significantly lower than the other two groups. On day 14, the negative and positive control groups displayed complete separation of the graft tissue from the host. Infiltration of inflammatory cells, predominantly lymphocytes, and the presence of plasma cells were observed in both groups. Numerous fibroblasts with elongated nuclei were also evident. In the positive control group, a reparative response characterized by the initiation of epithelial tissue formation was observed. In contrast, the treatment group exhibited a healthy epithelial layer and distinct connective tissue. Active fibroblasts, organized connective tissue containing collagen strands, and abundant vessels were present. Additionally, angiogenesis was evident in the treatment group, suggesting a favorable environment for graft survival. Notably, no signs of chronic graft rejection were observed in the treatment group (Figures 3 and 4).

*Real-time PCR*

**Figure 5:**
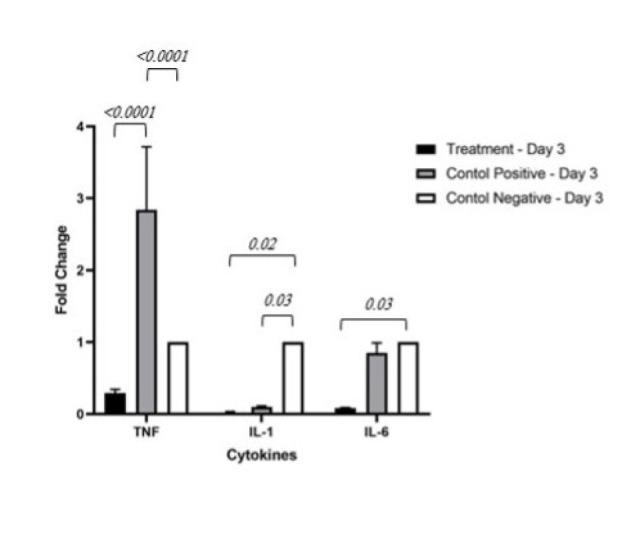
Comparison of inflammatory cytokine gene expression levels in mice on Day 3 among treatment, positive control, and negative control groups

**Figure 6:**
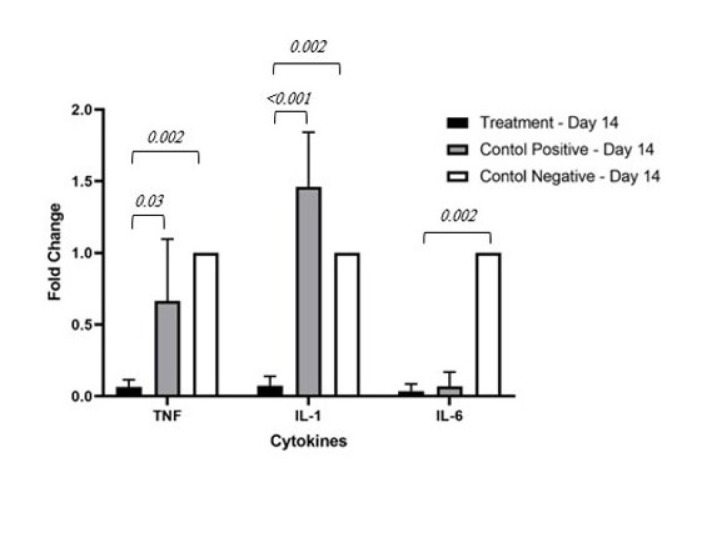
Comparison of inflammatory cytokine gene expression levels in mice on Day 14 among treatment, positive control, and negative control groups

The standard curve displays a correlation coefficient of 0.997 and a slope of -3.147. The melting curve analysis indicated the appropriate performance of the technique. A significant difference between all groups was observed, as demonstrated in Table 2. Specifically, the treatment group exhibited lower inflammatory cytokine gene expression levels than the positive and negative control groups (Figures 5 and 6). 

In summary, the results indicated that MSCs-derived exosomes exhibited potential therapeutic efficacy in reducing graft rejection symptoms. Visual observations and histopathological analysis demonstrated milder signs of graft rejection and a more favorable tissue environment in the treatment group compared to the negative and positive control groups. Additionally, real-time PCR analysis revealed a down-regulation of inflammatory cytokine gene expression in the treatment group, further supporting the beneficial effects of the administered extracellular vesicles. These findings suggest that the use of MSCs-derived exosomes holds promise for graft survival enhancement in transplantation settings.

## Discussion

Organ transplantation represents a highly efficient therapeutic approach for addressing numerous terminal diseases. Nevertheless, one of the foremost hurdles in transplantation is figuring out methods to ensure the enduring viability of allogeneic or xenogeneic grafts, such as preventing the rejection of transplants (4). Developing an effective means of suppressing immune rejection is among the crucial strategies for advancing clinical transplantation success (33). Exosomes are emerging as the most promising therapeutic elements among the bioactive substances released by MSCs. Research indicates that MSCs can address organ damage primarily through a paracrine-mediated pathway, even at low rates of MSC implantation. Due to their distinctive properties and effectiveness in various disease models, exosomes produced by MSCs have gained significant importance in the “paracrine hypothesis.” Moreover, MSC-derived exosomes are regarded as potential regulators of peripheral tolerance and immune responses (20).

As demonstrated by Helwa *et al.* (2017), the use of commercial kits for exosome extraction provides consistent particle quality and size distribution, indicating reliable isolation across different samples (34). A 2017 study further support the idea that commercial extraction kits yield adequately purified exosomes and are observed via electron microscopy (35). Thus, these kits streamline the exosome isolation process, ensuring high-quality results suitable for subsequent analyses (36).

Multiple studies have investigated the effects of exosomes derived from MSCs on immunomodulation. A study discovered that exosomes obtained from human umbilical cord MSCs, in conjunction with miR-181c, exhibited the ability to regulate immune responses in the context of burn injuries. The group that received these exosomes demonstrated a reduction in the expression levels of pro-inflammatory cytokines such as TNF-α and IL-1β, while the levels of anti-inflammatory cytokines like IL-10 increased. (37). Researchers showed that exosomes derived from bone marrow MSCs reduced pro-inflammatory factors (TNF-α and IL-1β), increased anti-inflammatory factors (TGF-β), increased levels of regulatory T cells (T-reg) and promoted Th1 to Th2 conversion in peripheral blood mononuclear cells. (38). A study presented a new method of using exosomes to deliver RNA molecules to dendritic cells (DCs). The RNA molecules are designed to reduce the expression of two factors that promote immune responses: HLA-G and mTOR. The authors showed that this method can make DCs less active and more tolerant and prevent the rejection of skin grafts in mice (39). A study investigated gene transfer through exosomes in islet transplantation and observed immune response suppression effects and improved graft survival (40). Another study found that exosomes derived from MSCs were effective in treating graft-versus-host disease (GVHD) in mice (41).

Following Pakyari *et al.* (2016), different mouse strains were used for donor and recipient groups to introduce immune response differences. This variation helps model graft rejection and healing more realistically, as using the same strain would lack immune variance and yield fewer representative results (24).

These studies collectively demonstrate the immunomodulatory effects of exosomes derived from MSCs and their potential to regulate inflammation and immune responses in various conditions, which is a confirmation of the results obtained in this study. In the immunological analysis of this study, conducted on the third day, the expression level of the IL-1β gene was significantly lower in the treatment group compared to the other groups. Consequently, it can be concluded that the activation of T helper cells is also much lower in this group. Therefore, the expression levels of IL-2 and INF-γ, as well as the cloning of B cells and subsequently the production of antibodies, and ultimately the activation of macrophages are lower in the treatment group (42). However, these changes are also valid for the group receiving dexamethasone. However, the intensity of immunomodulation in the exosome group seems higher than that of the positive control group. The expression of TNF-α in the treatment group has been significantly lower compared to the other groups, which justifies the low level of IL-6 in this group as well. Based on this, it can be expected that the treatment group has a reduced direct cytotoxic response on graft tissue, as well as a decrease in neutrophil activation and a decrease in the induction of endothelial adhesion molecules compared to the other groups. The low level of IL-6 in the treatment group can also indicate an increase in Treg activity (42).

The injection method and dosage in various studies range from 1 µg (41) to 800 µg, with different methods employed for administration (43). This diversity presents a significant challenge for our study. However, based on most of the literature, we opted for a preferred total dose of 200 µg delivered in two injections. The intraperitoneal injection method was chosen for its ease and reduced stress on the animals. 

Results similar to those on the third day are observed on the fourteenth day. The difference is that the expression level of TNF-α in the positive control group has become lower than in the negative control group. Generally, on this day as well, immunomodulatory effects such as a decrease in T helper activation, cloning of B cells, antibodies, neutrophil activation, and the induction of endothelial adhesion molecules were expected.

The analysis and comparison of this study’s macroscopic and histopathological results provide valuable insights into the immunomodulatory effects of MSCs-derived exosomes in transplantation. According to the results, exosomes have been able to achieve acceptable results in preventing solid graft rejection. These effects were clearly observed in the macroscopic results on day 3, but signs of graft rejection were observed in the macroscopic results on day 14. However, the intensity of these symptoms was much milder than that of other groups. Additionally, histopathological results also demonstrated the desirable effects of exosomes. In the histopathological findings, there were no signs of graft rejection on both days 3 and 14, and signs of graft survival, such as neovascularization, were observed in the exosome treatment group. Despite the lack of desirable macroscopic efficacy on day 14, the results obtained in this group were more promising compared to other groups. This indicates the potential for improving the working methods and utilizing new technologies and novel materials specifically developed for working with extracellular vesicles. By addressing these aspects, the quality of the results can be enhanced. This response could be influenced by various factors that may influence their efficacy. One important factor to consider is the dose and intervals of exosome administration. Optimizing the dosage and concentration of extracellular vesicles is crucial and requires more research. Additionally, the quality and function of exosomes, as well as the method of their extraction, can significantly impact the results (18). Other confounding factors, such as the non-isolation of FBS EVs, should also be taken into account (44). 

Therefore, it can be concluded that exosomes have had significantly more desirable effects in preventing graft rejection compared to the steroid drugs used as the positive control group in this study. An important point to note about the mentioned changes in cytokines is that these changes occur in most solid organ transplant surgeries (24, 42). Therefore, when exosomes can improve these changes in a skin graft, which is considered a solid organ, it can be inferred that they can also exert the effects mentioned in other solid organ transplantation, which requires more studies.

**Table 1:** Primer sequences used for Real-Time PCR for the mice genes (from the Origene company website)

Target templates	Primer	Sequence (5'->3')
beta-actin	Forward	CATTGCTGACAGGATGCAGAAGG
	Reverse	TGCTGGAAGGTGGACAGTGAGG
IL-1β	Forward	TGGACCTTCCAGGATGAGGACA
	Reverse	GTTCATCTCGGAGCCTGTAGTG
IL-6	Forward	TACCACTTCACAAGTCGGAGGC
	Reverse	CTGCAAGTGCATCATCGTTGTTC
TNF-α	Forward	GGTGCCTATGTCTCAGCCTCTT
	Reverse	GCCATAGAACTGATGAGAGGGAG

Numerous spherical bodies with relatively uniform and homogeneous structures can be seen in mice samples.

On day 3, the negative and positive control groups showed tissue browning and firm texture. The treatment group had no signs of graft rejection. On day 14, all groups showed signs of graft rejection. The negative and positive control groups had complete rejection with black tissue and firm texture. The treatment group had milder symptoms, such as tissue browning and semi-firm texture.symptoms, such as tissue browning and semi-firm texture.

Magnification is shown at ×4. Black triangles indicate the junctions of grafts. On Day 3, epithelial necrosis is evident in the negative control group, with visible adipose tissue and connective tissue strands beneath the hypodermis. In the positive control group, epithelial necrosis is also observed, along with brown pigmented regions, which may indicate hemosiderin or hematin accumulation. In the exosome-treated group, there is evidence of peripheral necrosis near the graft junction, and adipose tissue is clearly visible. On Day 14, in the negative control group, the connective tissue has fully separated, with new epithelial growth from the right side and some remaining necrotic tissue centrally. In the dexamethasone-treated group, extensive necrosis of connective tissue is seen, with new connective tissue formation underneath. The epithelial tissue appears intact in the exosome-treated group, and organized connective tissue has formed beneath it.

Magnification is shown at ×4 and ×10. – a: Epithelial necrosis is evident, adipose tissue is observed, and the muscular layer is wholly necrotized. The infiltration of inflammatory cells is clearly visible. b: Epithelial necrosis is more prominently evident. Additionally, circular-shaped areas indicate necrotic hair follicles; and within them, pyknotic nuclei and eosinophilic cytoplasm are visible. c, d: It is observed that the necrotic graft tissue has wholly separated from the host tissue. Additionally, the connective tissue shows a significant number of fibroblast cells with elongated nuclei, lymphocytes, plasma cells, and a limited number of neutrophils. e, f: Necrotic epithelium, infiltration of polymorphonuclear inflammatory cells, and adipose tissue are observed. g, h: Host reparative tissue is observed. The predominant cells are lymphocytes and blood vessels are forming. i: The epithelium appears intact, but there is an underlying hemorrhage. The presence of inflammatory cells is also observed, albeit in a milder form compared to the other two groups. j: In this section, it also appears that the epithelium is intact, and hemorrhage is still visible. Additionally, there is a presence of a limited number of neutrophils and mononuclear inflammatory cells. k: It appears that the epithelium is intact, and in the center, a circular structure is seen, a hair follicle. Additionally, infiltration of inflammatory cells is evident. In the lower part, connective tissue is also observed. l: A highly vascularized connective tissue is observed, which is one of the crucial factors in the rejection or survival of the graft. Some fibroblast cells with delicate collagen strands are also seen.

**Table 2:** *P *value to compare the statistical difference between all mice groups in different time points

Day	Groups	*P-value*	Summary
3	Treatment vs Control Positive vs Control Negative	0.007	*Significant*
14	Treatment vs Control Positive vs Control Negative	0.0047	*Significant*

There is a significant difference in all time points between groups

This bar graph illustrates the fold change in expression of the inflammatory cytokines TNF, IL-1, and IL-6 at Day 3 post-treatment. The treatment group (black bars) shows significantly lower expression levels of these cytokines compared to the positive control group (gray bars) and negative control group (white bars), with *P*-values denoted above each comparison. Lower cytokine expression in the treatment group indicates a reduced inflammatory response.

This bar graph illustrates the fold change in expression of the inflammatory cytokines TNF, IL-1, and IL-6 at day 14 post-treatment. The treatment group (black bars) again shows significantly reduced cytokine expression compared to both control groups, with specific *P*-values provided for each cytokine comparison. The sustained reduction in inflammatory cytokine levels in the treatment group over time suggests a prolonged anti-inflammatory effect.

## Conclusion

The findings of this study shed light on the immunomodulatory effects of MSC-derived exosomes in solid organ transplantation and their potential to enhance graft survival while mitigating graft rejection. Despite facing certain limitations, the results clearly demonstrate the desirable effects of exosomes in histopathology and immunology in both short- and long-term survival, particularly in preventing acute graft rejection. To further optimize the use of exosomes, additional research is required to determine the ideal dosage and concentration for long-term benefits. Moreover, considering the immunomodulatory effects observed in this study and corroborated by previous research, it is reasonable to anticipate similar positive outcomes in the context of other solid organ transplantation settings. Ultimately, exosomes hold immense promise as a potential therapeutic approach to prevent graft rejection in the future. However, realizing this potential hinges upon optimizing and standardizing the methods for isolating and characterizing exosomes in a clinical setting and establishing their effective dosage. Additionally, comprehensive studies are necessary to assess the cost-effectiveness of exosomes compared to alternative methods, allowing for informed decisions regarding commercialization and further clinical investigations.

## Funding

The article lacks any sources of funding that require declaration.

## Data Availability

We declare that the data and materials supporting the findings of this study are available upon request and will be provided by the corresponding author.

## Declaration

During the preparation of this work, the authors employed the services of ChatGPT, an advanced language model developed by OpenAI, to enhance the quality and coherence of the text. Leveraging this tool for paraphrasing and rewriting, the authors diligently reviewed and edited the content, assuming full responsibility for the final publication.
