# Supplementary material for: Preventing the rejection of skin allografts by immunomodulatory and regenerative effects of exosomes derived from bone marrow mesenchymal stem cells in mice
Source: Iran J Basic Med Sci. 2025;28(4):469–76. doi: 10.22038/ijbms.2025.82564.17841 (PMC11831755; doi:10.22038/ijbms.2025.82564.17841)
Supplement: Supplementary file 1 [file ijbms-28-469-s001.pdf]

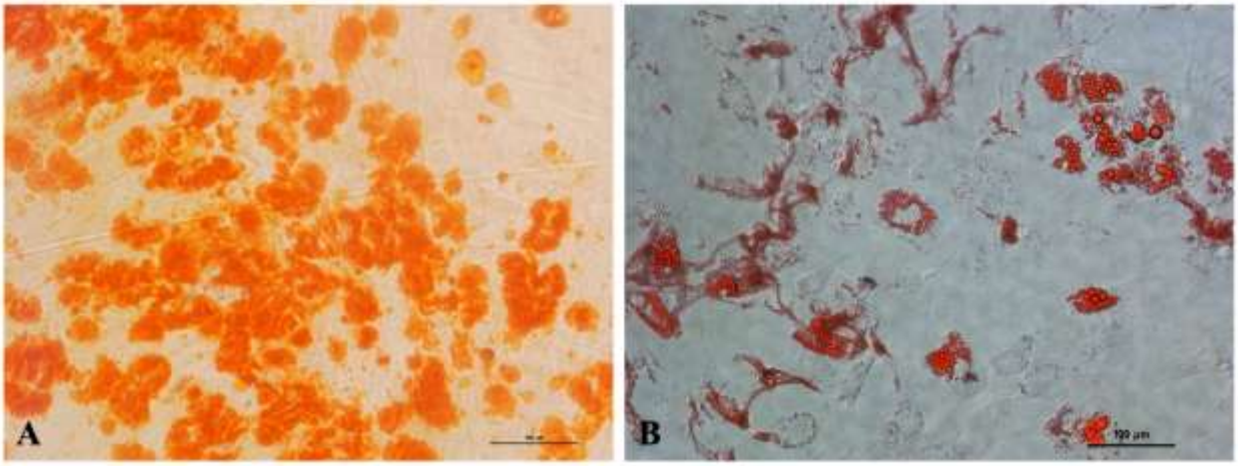

Figure 1. (**A**) Representative photomicrograph of mesenchymal stem cells (MSCs) differentiated into osteogenic lineage and stained with Alizarin Red S. The staining highlights the calcium deposits in the extracellular matrix, indicative of successful osteogenic differentiation. The red-orange staining indicates mineralized nodules. Scale bar: 100 µm. - (**B**) Representative photomicrograph of MSCs differentiated into adipogenic lineage and stained with Oil Red O. The staining visualizes lipid droplets accumulated within the differentiated adipocytes, confirming adipogenic differentiation. Lipid vacuoles appear as red-stained droplets. Scale bar: 100 µm.

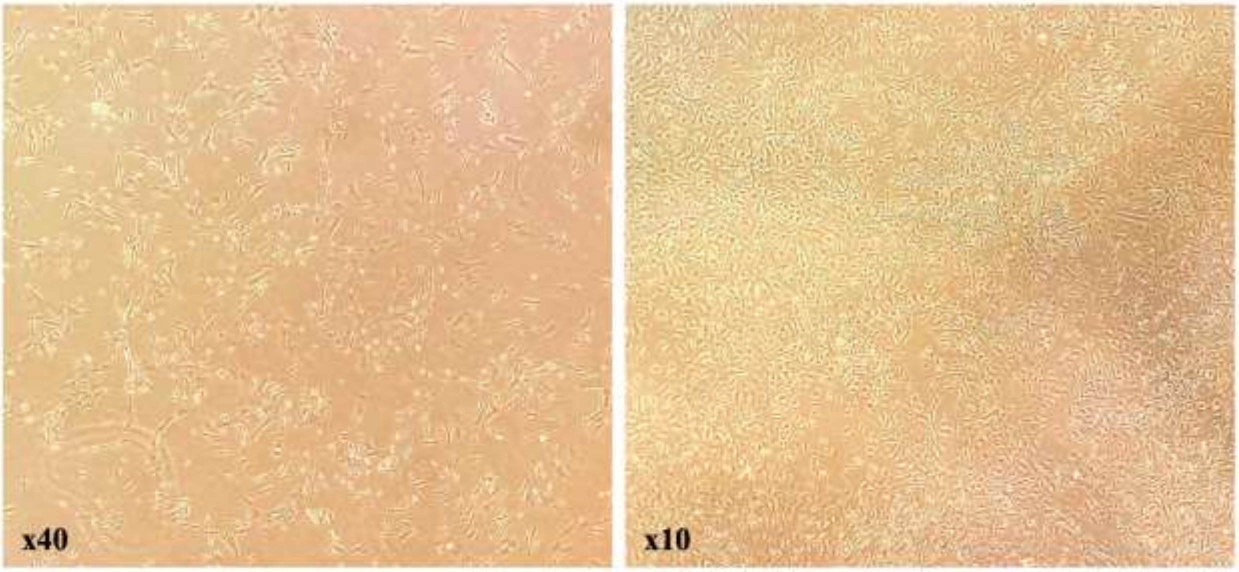

Figure 2. Spindle-shaped mesenchymal stem cells with a small cell body and elongated cellular processes were observed in the culture flask. The cell body contains a large, round, prominent nucleus with a clear appearance. Images were captured at 10x and 40x magnifications using an inverted light microscope. Both images correspond to cells at passage three.

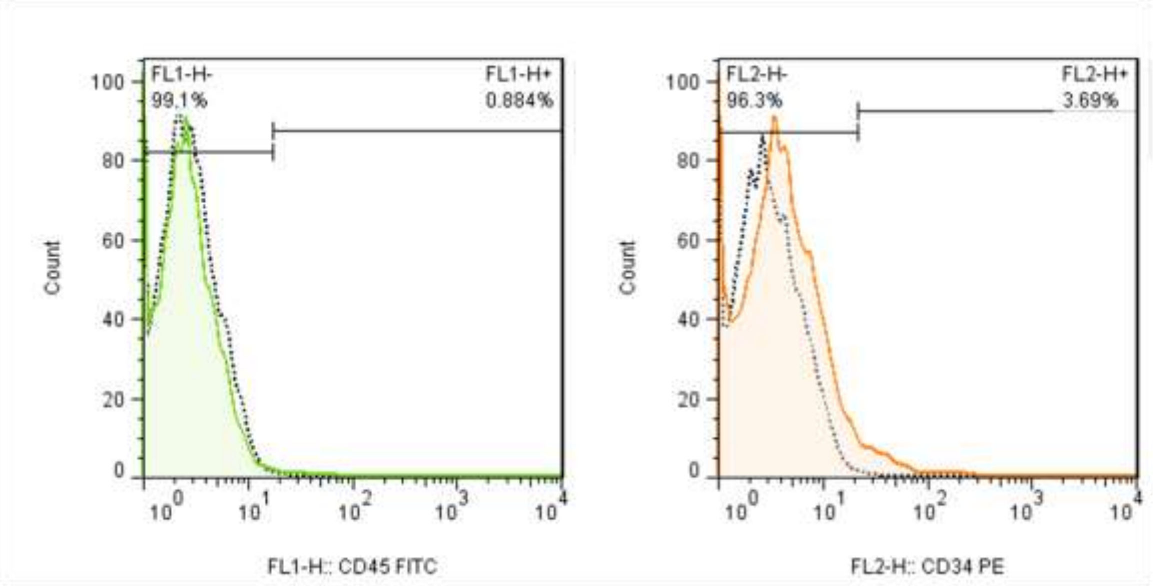

Figure 3. Phenotyping of mesenchymal stem cells was performed using flow cytometry, confirming the absence of CD34 (right) and CD45 (left) markers. The results indicated that over 90% of mesenchymal stem cells lacked the hematopoietic markers CD34 and CD45.

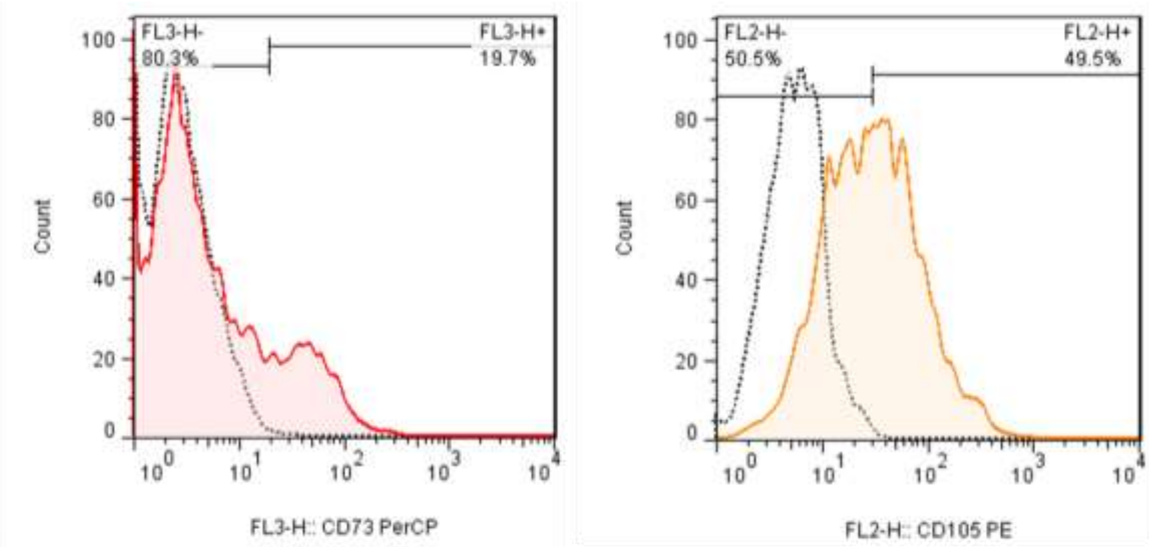

Figure 4. Phenotyping of mesenchymal stem cells was conducted using flow cytometry, focusing on the positive markers CD105 (right) and CD73 (left). The mesenchymal stem cell surface markers CD73 and CD105 were present in 20% and 50% of the cells, respectively.

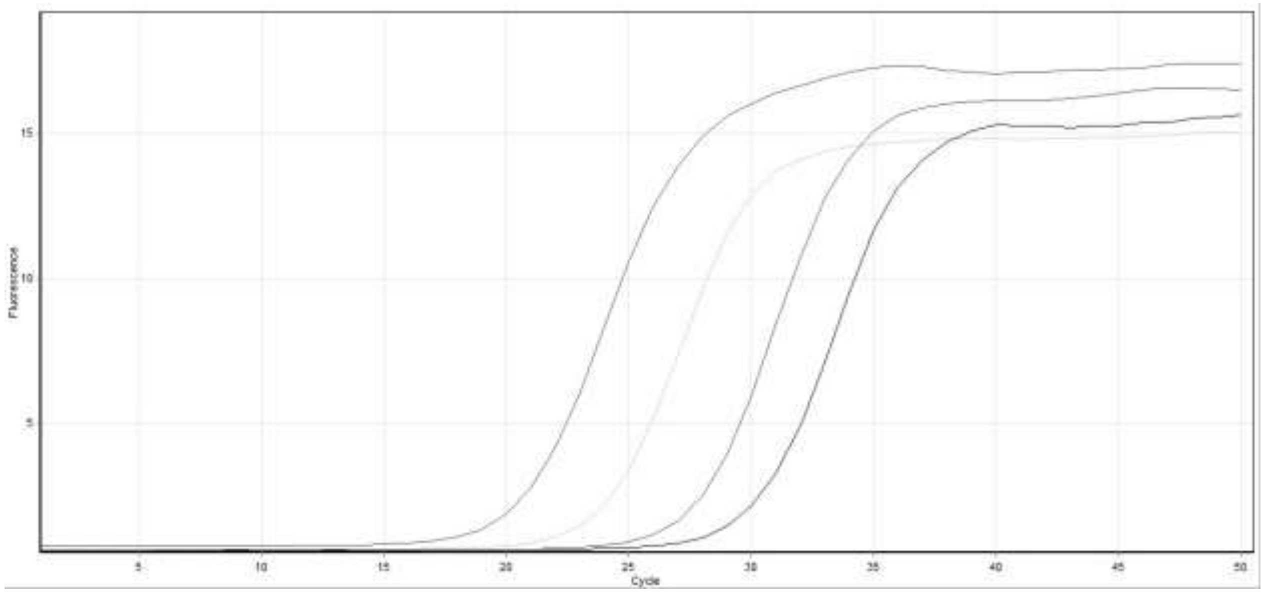

Figure 5. displays proliferation curves for inflammatory cytokines in standard samples at specified concentrations. The curves represent four different concentrations of beta-actin: 0.1, 0.01, 0.001, and 0.0001.

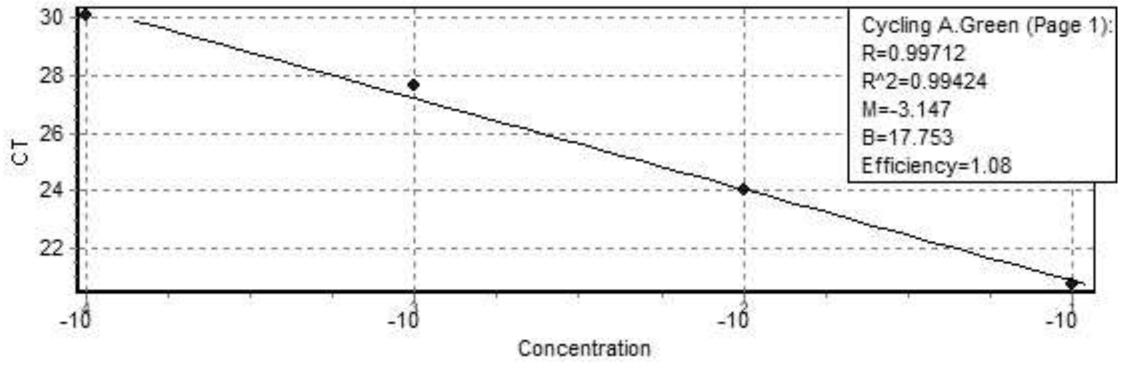

Figure 6. Following the analysis, CT values were calculated for each standard concentration, and a standard curve was plotted, as shown in this figure. The correlation coefficient of the data was 0.997, with a slope of -3.147, indicating that the technique adheres to established standards. Furthermore, the calculated efficiency of this technique was 1.08, representing an acceptable value.
